# Urinary Neutrophil Gelatinase-Associated Lipocalin (NGAL) and proteinuria predict severity of acute kidney injury in Puumala virus infection

**DOI:** 10.1186/s12879-015-1180-9

**Published:** 2015-10-27

**Authors:** Hanno Bunz, Peter Weyrich, Andreas Peter, Dorothea Baumann, Otto Tschritter, Martina Guthoff, Robert Beck, Gerhard Jahn, Ferruh Artunc, Hans-Ulrich Häring, Nils Heyne, Robert Wagner

**Affiliations:** Department of Internal Medicine, Division of Endocrinology, Diabetology, Nephrology, Vascular Disease and Clinical Chemistry, University Hospital of the Eberhard Karls University, Tübingen, Germany; Institute for Diabetes Research and Metabolic Diseases of the Helmholtz Centre Munich at the University of Tübingen (IDM), Tübingen, Germany; German Center for Diabetes Research (DZD), Tübingen, Germany; Institute of Medical Virology and Epidemiology of Viral Diseases, Eberhard Karls University, Tübingen, Germany

**Keywords:** Hantavirus, Nephropathia epidemica, Acute kidney injury, NGAL, Neutrophil Gelatinase-Associated Lipocalin, Puumala virus infection, Hemorrhagic fever with renal syndrome

## Abstract

**Background:**

Nephropathia epidemica (NE) is a mild form of hemorrhagic fever with renal syndrome (HFRS) that is caused by the Puumala virus. Periodic outbreaks have been described in endemic areas, with a substantial number of previously healthy individuals developing acute kidney injury (AKI). There is a considerable diversity in the clinical course of the disease, and few patients require renal replacement therapy.

**Methods:**

We tested whether urinary neutrophil gelatinase associated lipocalin (uNGAL), urine albumin/creatinine ratio (uACR), urine protein/creatinine ratio (uPCR), urine dipstick protein, C-reactive protein, procalcitonin, leukocyte and platelet count, determined on admission to the hospital, can predict the severity of AKI. Sixty-one patients were analyzed during admission in the emergency department.

**Results:**

The variables most strongly associated with peak plasma creatinine concentration were uNGAL (β = 0.70, *p* <0.0001), uPCR (β = 0.64, *p* = 0.001), uACR (β = 0.61, *p* = 0.002), and dipstick proteinuria (β = 0.34, *p* = 0.008). The highest AUC-ROC to predict stage 3 AKI according to the acute kidney injury network’s (AKIN) classification was seen for uNGAL (0.81, *p* = 0.001).

**Conclusion:**

uNGAL accurately predicts the severity of AKI in NE. This could help emergency room physicians predict disease severity and allow for initial risk stratification.

**Electronic supplementary material:**

The online version of this article (doi:10.1186/s12879-015-1180-9) contains supplementary material, which is available to authorized users.

## Background

Nephropathia epidemica (NE) is a common cause for acute kidney injury (AKI) in otherwise healthy persons living in endemic regions. Although it is a relatively mild form of hemorrhagic fever with renal syndrome, the frequency of periodic outbreaks has increased over the last years, and the increasing number of infections with high rates of hospitalization makes it an emerging public health threat [1]. NE is caused by the Puumala virus (PUUV), a member of the Hantavirus genus. The virus is spread through aerosolized excrements of distinct rodents, the bank voles (Myodes glareolus), while the rodents themselves are probably asymptomatic carriers [2]. In 2012 there were 2824 cases of NE reported in Germany, representing the highest incidence since the introduction of a mandatory nationwide register in 2006 [3].

PUUV infections manifest after an incubation period of 2–4 weeks starting with non-specific flu-like symptoms such as fever and headache. A considerable proportion of infections may have a subclinical course or only cause light symptoms [4]. Acute kidney injury (AKI), often introduced by severe flank pain and oliguria, is a potentially serious consequence which can lead to fluid retention, hyperkalemia and the need for renal replacement therapy in some cases [5, 6]. We recently showed that the use of non-steroid anti-inflammatory drugs in the acute phase is associated with a more severe disease [7]. Up to now, there is no established method to predict the severity of AKI upon onset of symptoms or admission. Therefore, patients with clinically suspected and/or serologically proven PUUV infections are usually hospitalized for observation. A marker reliably predicting the severity of AKI would be of clinical relevance, since it could aid emergency-room physicians in triaging patients with suspected or confirmed NE.

In this study, we tested the association of plasma and urinary laboratory parameters with disease severity in patients with NE who have been admitted to our nephrology unit through the emergency room (ER) of the university hospital. The investigated parameters comprised leukocyte and platelet count, plasma C-reactive protein (CRP) and procalcitonin (PCT), plasma sodium, urinary protein-to-creatinine ratio (uPCR), urinary albumin-to-creatinine ratio (uACR), urine dipstick protein (semiquantitative measurement), urinary neutrophil gelatinase associated lipocalin (uNGAL), the ratio of NGAL to creatinine in urine (uNGAL/uCrea). Neutrophil gelatinase associated lipocalin (NGAL) in urine or plasma is an emerging marker of AKI, which has been shown to correlate with severity of AKI [8]. NGAL is a small protein in mono and multimeric forms with a size of approximately 25-kDa [9]. It is expressed in neutrophils and human epithelia especially of liver and kidney tissue.

Because of its size, NGAL is filtered in the glomerulus and reabsorbed in the proximal tubule. Urinary NGAL can discriminate between acute allograft rejection and AKI of other causes [10].

## Methods

### Study population

We retrospectively identified 61 patients with NE who were admitted to our nephrology unit through the ER of the university hospital in 2012. The Ethics Committee of the Tübingen University Medical Faculty has waived the need for informed patient consent for this retrospective analysis (Reference: 049/2015R). Anonymized data on anthropometric parameters, duration of hospital stay, quantity of maximum urinary output and weight change, defined as the difference between the highest and lowest recorded weight during hospital stay, were extracted by chart review. The assessed laboratory parameters comprised leukocyte count, platelet count, CRP, PCT, initial plasma creatinine, peak plasma creatinine during hospital stay, uPCR, uACR, dipstick proteinuria, uNGAL, uNGAL/uCrea and serologic status. PUUV infections were confirmed by IgM and IgG-capture ELISA and in doubt proven by Western Blot. A positive reaction in IgM-ELISA was necessary for study-inclusion.

### Laboratory analysis

Laboratory measurements were performed in the central laboratory of the university hospital as ordered by attending physicians. Blood cell and platelet counts were determined on the ADVIA 2120 hematology analyzer. Semiquantitative proteinuria (“urine dipstick protein”) was determined using the iChemVELOCITY urinalysis system (Iris Diagnostics, Beckman Coulter, Krefeld, Germany). Urinary albumin levels were measured nephelometrically (BN Prospec Nephelometer). Urinary protein was determined using the benzethonium chloride method (Roche Diagnostics, Mannheim Germany) and CRP was determined using a wide-range latex-enhanced immunturbidimetric assay. Plasma and urine creatinine was measured enzymatically using the creatinase method and urinary concentrations of NGAL were determined using a particle-enhanced turbidimetric immunoassay (BioPorto Diagnostics, Gentofte, Denmark). Plasma sodium and all other measurements above were performed on an ADVIA 1800 clinical chemistry analyzer (all instruments from Siemens Healthcare Diagnostics, Eschborn Germany).

PCT concentrations were determined using the BRAHMS KRYPTOR Time Resolved Amplified Cryptate Emission Immunoassay system (Thermo Scientific, Hennigsdorf, Germany).

### Calculations and statistical analysis

The severity of AKI was evaluated according to the staging system devised by the Acute Kidney Injury Network [11]. The increase of creatinine was used as the main marker of the severity of AKI (increase of creatinine to equal or more than 3-fold of the baseline creatinine value). None of the patients had previously known chronic kidney disease. Therefore, basal creatinine values were estimated from normal glomerular filtration rates that are normal for sex and age, standardized on body surface area (1.73 m^2^) [12]. To calculate normal plasma creatinine levels, creatinine levels were backtraced from the CKD-EPI equation (Chronic Kidney Disease Epidemiology Collaboration) by solving the equation for this variable.

Furthermore, we calculated length of hospital stay and retrieved data on body weight and urine output from analyzed charts. Change of body weight during hospital stay was calculated by subtraction of the lowest measured weight from the highest measured weight.

For statistical analysis in this study, we decided to use uNGAL raw data including quantitative values below the limit of quantification of 25 ng/ml, defined by a coefficient of variation ≤20 %. In patients with no detectable uNGAL concentration, the value of 1 ng/ml was used instead of zero, to enable log-transformation.

Results of the semiquantitative urine protein measurement approximately correlate with albuminuria as follows (+) 0.1 ~ 0.5 g/L, + 0.5 g/L, ++ 1 g/L ~ 3 g/L and +++ over 3 g/L.

Univariable linear regression analyses were used to assess the association of possible predictors with peak plasma creatinine. In multivariable linear regression analyses, predictors were adjusted for sex, age and BMI. Estimates are given as standardized betas (β_std)_.

Parameters with skewed distributions were log-transformed prior to analyses.

Receiver operating characteristic (ROC) curves were used to assess the discriminative power of investigated predictor variables of severe AKI. All statistical calculations were performed with JMP 11.0 (SAS, Cary, NC, USA).

## Results

We identified 61 patients with serologically confirmed NE who were admitted to our nephrology unit during 2012. None of the patients required hemodialysis. Patient characteristics with descriptive data on investigated biomarkers and outcome variables are shown in Table 1 (for distribution of uNGAL levels, see Additional file 1: Figure S1).

**Table 1:** Demographic, clinical and laboratory parameters of the study population (*N* = 61)

	Median	IQR
Age (years)	45	33, 52
Sex (m/f)	45/16	
BMI (kg/m^2^, *n* = 58)	24.5	22.5, 28.6
Days between onset of symptoms and hospital admission	6	4, 7
Length of inpatient stay (days)	7	5, 9
Span of body weight change during hospital stay (kg, *n* = 56)^a^	4.2	2.3, 5.8
Highest urine output per 24 h (ml, *n* = 56)	5850	4500, 8300
Leukocyte count at admission (1/μl)	8680	6685, 10725
Platelet count at admission (thousand/μl)	109	71, 178
Plasma Sodium (mmol/l)	135	132, 139
C-reactive protein at admission (mg/dl, *n* = 60)	6.3	3.9, 9.6
Procalcitonin at admission (ng/ml, *n* = 19)	1.27	0.9, 1.9
Estimated baseline creatinine (μmol/l)	86	82, 89
Plasma creatinine at admission (μmol/l)	221	106, 344
Peak plasma creatinine (μmol/l)	344	185, 618
Patients with AKIN stage 0–2/3	23/38	
Urinary protein/creatinine ratio (mg/g, *n* = 23)	1492	552, 10303
Urinary albumin/creatinine ratio (mg/g, *n* = 24)	1007	223, 8440
Urine dipstick protein (neg/(+)/+/++/+++, *n* = 59)	2/6/17/28/6	
Urinary NGAL (ng/ml, *n* = 55)	83	31, 189
Urinary NGAL/creatinine ratio (ng/mg, *n* = 49)	137	43, 371

^a^highest documented weight – lowest documented weight

### Peak plasma creatinine concentration

**Fig. 1:**
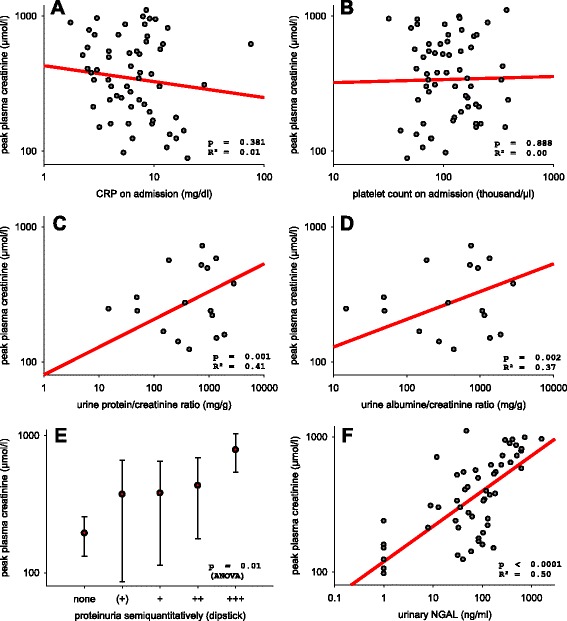
**a**–**f** Correlation of possible predictor variables. Correlation of possible predictor variables with peak plasma creatinine concentration during hospital stay. In panels (**a**, **b**, **c**, **d** and **f**) both axes are log-scaled. Only the y-axis is log-scaled in panel (**e**). Panel (**e**) shows category means +− SDs

While platelet count, CRP and procalcitonin did not show significant correlations with peak plasma creatinine concentration, uNGAL and markers of proteinuria (urine dipstick proteinuria, uACR and uPCR) were associated with it (see Table 2 and Fig. 1). Controlling for the potential confounders sex, age and BMI did not relevantly change these associations (see Table 3). The use of the normalized variable uNGAL/uCrea instead of uNGAL did not confer further improvement in the β-estimate.

**Table 2:** Correlation of laboratory markers with variables of disease severity; a. univariate

	Peak plasma creatinine		Duration of hospital stay		Weight cycling		Peak urine output	
	R^2^	β_std_ (p)	R^2^	β_std_ (p)	R^2^	β_std_ (p)	R^2^	β_std_ (p)
Leukocyte count	0.08	0.28 (0.026)	0.03	0.17 (0.185)	<0.01	−0.0 (0.99)	0.01	−0.09 (0.489)
Platelet count	0.00	0.02 (0.888)	0.08	−0.29 (0.024)	0.06	−0.26 (0.06)	0.03	0.17 (0.214)
Plasma sodium	0.11	−0.33 (0.010)	0.09	−0.30 (0.021)	0.09	−0.29 (0.03)	0.00	−0.02 (0.902)
C-reactive protein	0.01	−0.12 (0.381)	0.02	0.16 (0.236)	<0.01	−0.05 (0.72)	0.09	−0.30 (0.029)
Procalcitonin	0.12	0.35 (0.145)	0.20	0.45 (0.055)	0.09	0.30 (0.22)	0.04	−0.19 (0.442)
Urine protein/creatinine	0.41	0.64 (0.001)	0.06	0.25 (0.256)	0.05	0.23 (0.32)	0.00	−0.03 (0.912)
Urine albumine/creatinine	0.37	0.61 (0.002)	0.15	0.38 (0.065)	0.15	0.38 (0.07)	0.00	−0.02 (0.934)
Urinary NGAL	0.50	0.70 (<0.0001)	0.31	0.56 (<0.001)	0.09	0.31 (0.03)	0.00	0.00 (0.999)
Urinary NGAL/creatinine	0.47	0.69 (<0.0001)	0.22	0.47 (0.001)	0.09	0.31 (0.03)	0.00	0.03 (0.837)

**Table 3:** Correlation of laboratory markers with variables of disease severity; b. multivariate (adjusted for sex, age and BMI)

	Peak plasma creatinine		Duration of hospital stay		Weight cycling		Peak urine output	
	R^2^	β_std_ (p)	R^2^	β_std_ (p)	R^2^	β_std_ (p)	R^2^	β_std_ (p)
Leukocyte count	0.08	0.25 (0.068)	0.03	0.12 (0.383)	0.07	0.005 (0.97)	0.10	−0.12 (0.379)
Platelet count	0.03	−0.10 (0.456)	0.18	−0.41 (0.002)	0.12	−0.22 (0.09)	0.13	0.20 (0.129)
Plasma sodium	0.12	−0.32 (0.019)	0.13	−0.35 (0.010)	0.18	−0.35 (0.013)	0.09	−0.03 (0.839)
C-reactive protein	0.02	−0.05 (0.742)	0.10	0.29 (0.030)	0.09	−0.03 (0.85)	0.15	−0.26 (0.051)
Procalcitonin	0.38	0.62 (0.034)	0.41	0.80 (0.007)	0.12	0.42 (0.26)	0.05	−0.25 (0.446)
Urine protein/creatinine	0.44	0.70 (0.002)	0.13	0.32 (0.194)	0.11	0.31 (0.23)	0.08	0.04 (0.855)
Urine albumine/creatinine	0.40	0.67 (0.002)	0.19	0.43 (0.054)	0.20	0.40 (0.09)	0.09	0.08 (0.731)
Urinary NGAL	0.51	0.71 (<0.0001)	0.28	0.52 (<0.0001)	0.17	0.31 (0.0385)	0.08	0.01 (0.971)
Urinary NGAL/creatinine	0.47	0.68 (<0.0001)	0.17	0.41 (0.005)	0.17	0.31 (0.0385)	0.07	0.08 (0.605)

To test whether the association between uNGAL and peak plasma creatinine concentration is independent from proteinuria, we fitted uNGAL to peak plasma creatinine concentration in multivariable linear regression models also controlling for uACR or uPCR. Adding covariables of albuminuria (p_ACR_ =0.03) or proteinuria (p_PCR_ = 0.04) did not abolish the significant association of uNGAL and peak plasma creatinine (p_NGAL_ = 0.002 in both models). Actually, the effect size estimate for uNGAL was numerically substantially higher than for uACR (β_std_ = 0.55 vs 0.35) or uPCR (β_std_ = 0.57 vs 0.35) in these combined models.

When testing the association between uNGAL and peak plasma creatinine concentration with additional adjustment for sex, age, BMI and uACR, uNGAL maintained its strong association. Even the association between uNGAL and duration of hospital stay was not affected (see Table 4).

**Table 4:** Correlation of laboratory markers with variables of disease severity; c. multivariate models of peak plasma creatinine concentration and duration of hospital stay (adjusted for sex, age, BMI, uACR, uNGAL)

	Peak plasma creatinine		Duration of hospital stay	
	R^2^	β_std_ (p)	R^2^	β_std_ (p)
Sex	0.64	−0.10 (0.514)	0.53	−0.34 (0.07)
Age		−0.06 (0.682)		0.39 (0.03)
BMI		0.04 (0.802)		−0.30 (0.10)
Urine albumine/creatinine		0.37 (0.049)		0.27 (0.18)
Urinary NGAL		0.56 (0.004)		0.49 (0.02)

### Acute kidney injury, AKIN stage 3

uNGAL and markers of proteinuria significantly and relevantly predicted a rise of plasma creatinine consistent with AKIN stage 3 (see Table 5, also see Additional file 1: Figure S2). The highest AUC-ROC was shown for uNGAL. Data in the ROC-table (see Additional file 1: Table S1) show that taking an NGAL cutoff of >130 ng/ml achieves a high specificity (specificity 91 %) and a high positive predictive value (90 %) for stage 3 AKI. A cutoff of ~30 ng/ml yielded a relatively high sensitivity for developing stage 3 AKI (sensitivity 90 %, negative predictive value = 75 %) (see Table 6).

**Table 5:** Odds ratio (OR) for developing AKIN stage 3 acute kidney injury

	OR	Lower CI	Upper CI	*p*	AUC-ROC
Urinary protein/creatinine ratio	4.71	1.04	46	0.04	0.71
Urinary albumin/creatinine ratio	4.11	1.33	21.68	0.01	0.75
uNGAL	5.97	2.35	21.65	0.001	0.80

Odds ratio (OR) for developing AKIN stage 3 acute kidney injury per 10-fold increase in urine protein/creatinine ratio, urine albumin/creatinine ratio and urinary NGAL

**Table 6:** NGAL cutoff

uNGAL (ng/ml)	<30^a^	30 – 130	>130^b^
uACR (mg/g)	<200^a^	200–2000	>2000^b^
Risk of severe acute kidney injury	low	intermediate	high

An example for urinary neutrophil gelatinase associated lipocalin (uNGAL) and uACR (urine albumin creatinine ratio) cutoffs to facilitate clinical decision-making in NE. Severe AKI is classified as an increase of plasma creatinine to >= 3x of baseline (AKIN stage 3)

^a^cutoff with high sensitivity (>90 %)

^b^cutoff with high specificity (>90 %)

### Other clinical variables of disease severity

Among the investigated clinical variables, uNGAL was significantly associated with hospital stay and weight change during hospital stay (see Table 2). Interestingly, platelet counts at admission associated with the length of hospital stay (see Table 2) but not with peak creatinine. The association between platelet count and hospital stay was stronger after controlling for sex, age and BMI (see Table 3), suggesting a negative confounding by sex, age and BMI. However, none of the covariates sex, age, and BMI associated with length of hospital stay in univariate analyses. Out of the other investigated biomarkers, plasma sodium, CRP (after adjustment only) and dipstick proteinuria associated with length of hospital stay. There was a negative association of CRP with peak polyuria, which may have been confounded by sex, age and BMI.

## Discussion

In this study, we provide first evidence that uNGAL, as a point of care variable determined in emergency room (ER) patients having symptoms compatible with PUUV, is a good predictor of the severity of AKI due to PUUV infection. Although markers of proteinuria (uACR, uPCR and semiquantitative proteinuria determined by dipstick test) also correlated with the severity of AKI, these associations were weaker and uNGAL preserved a considerably stronger association with peak creatinine in models adjusted for uACR or uPCR. The highest discriminatory power for severe AKI, defined as AKIN stage 3, among the investigated variables was observed for uNGAL.

Our study delivers a proof-of-principle that predicition of disease severity is feasible in NE. Most patients with NE present with unspecific flu-like symptoms such as headache and fever, but often develop severe flank pain and oliguria soon afterwards. Laboratory analysis usually demonstrates thrombocytopenia and elevated plasma creatinine concentration at this stage. However, creatinine is a tardy and unreliable marker of acute kidney injury. Striking differences in peak creatinine concentration and associated problems of AKI such as electrolyte disturbances and uremic symptoms are observed in patients. Weight change during disease may indicate fluid retention and could therefore be an additional marker of disease severity reflecting capillary leakage [13, 14].

In this study, we show that uNGAL, and to a lesser degree markers of proteinuria, are able to predict severity of AKI. Severe AKI in turn can be associated with complications and the need for intensive monitoring of fluid- and electrolyte status. In the case of uNGAL, its associations with length of hospital stay and weight change during hospital stay further underlines its role in predicting disease severity. Thus, determination of uNGAL in patients with suspected NE right upon first presentation could guide the clinician’s decision about hospital admission. Our data suggest that an uNGAL >130 ng/ml (positive predictive value 90 %, cf. Table 5) is associated with severe AKI prompting hospital admission for further monitoring. In contrast, patients with low NGAL (<30 ng/ml) are at a relatively low risk for severe AKI, and their care could be continued in an outpatient setting. Adjustment for urinary creatinine did not improve the correlation, as also shown in a previous work [10].

Whereas thrombocytopenia also associated with length of hospital stay in our study, it was not correlated with other variables of disease severity. Since thrombocytopenia is typical in early phases of NE, this association is probably confounded by the time of presentation in the ER relative to disease phase. Patients who tended to present at an earlier disease phase would have lower platelet counts and would therefore have to be observed longer until resolution of AKI. Thrombocytopenia, however, could prove to be a better predictor of disease severity if determined early in the disease course, as shown by Rasche et al. [15].

The underlying pathomechanisms of AKI in PUUV infection is not fully understood. Although the virus primarily replicates in endothelial cells, it does not cause major cytotoxicity or cell death [2]. Rather, it seems to transiently disrupt the integrity of cell-to-cell junctions in renal podocytes, tubular epithelial and glomerular endothelial cells [16]. However, the contrast of clear interstitial edema and occasional tubular damage with relatively spared glomeruli seen in renal biopsy specimens suggests that the majority of renal pathology is due to tubulointerstitial injury [17–19]. NGAL predominantly accumulates in epithelial cells of proximal tubules where it is thought to play a role in induction of reepithelialization [20, 21]. Therefore, the observed strong association of uNGAL with peak plasma creatinine concentration, which was independent of proteinuria, supports the predominantly tubulointerstitial injury in NE in two ways. NGAL produced by neutrophil cells is freely filtrated and reabsorbed at proximal tubular sites [22]. Hence, proximal tubular injury would result in increased NGAL excretion by reduced tubular reabsorption. Furthermore, NGAL mRNA expression and production is upregulated in the distal convoluted tubule and the collecting duct during tubular stress [23]. These mutually enhancing effects and the marked tubulointerstitial injury in NE [17] could explain the strong association of uNGAL with the severity of AKI.

Several other biomarkers have previously been associated with the severity of NE. These comprise plasma markers such as interleukin-6 [24], indoleamin 2,3-dioxygenase [25], pentraxin-3 [13], cell-free DNA [26] and the urinary biomarker GATA-3 [27]. One study found that the maximum leukocyte count, which has been seen 3–6 days after disease onset, correlated with fold-change in plasma creatinine [27]. As to our knowledge, association of NGAL and simple urine biomarkers such as uACR or uPCR with outcome variables has not been investigated before. uNGAL determined in the ER upon first presentation of the patient seems to be the clinically most relevant and robust predictor, which seems to be independent from disease phase and time of onset of symptoms.

The single most important limitation of our study is its retrospective aspect. In a university hospital setting, we also note that the patients in our cohort were admitted at a relatively late disease phase (median 6 days after the onset of first symptoms), which potentially explains why established early markers of disease severity such as thrombocytopenia did not perform well.

Nevertheless, our robust findings support the notion that uNGAL allows for prediction of severity of AKI, enabling a risk stratification of these patients upon first presentation in the ER for suspected NE. Future trials have to prospectively validate these results.

## Conclusion

Urinary NGAL, determined upon hospital admission accurately predicts severity of acute kidney injury in Puumala virus infection and allows for early risk stratification in these patients. Further trials are required to validate these findings prospectively.

## Additional file

Additional file 1:
**NE-NGAL BMC supp. data rev 2.0.pptx** (PPTX 161 kb)

## Abbreviations

AKI: Acute kidney injury
AKIN: Acute kidney injury network
CKD-EPI: Chronic kidney disease epidemiology collaboration
CRP: C-reactive protein
ER: Emergency room
NE: Nephropathia epidemica
PCT: Procalcitonin
PUUV: Puumala virus
ROC: Receiver operating characteristic
uACR: Urinary albumin creatinine ratio
uNGAL: Urinary Neutrophil Gelatinase-Associated Lipocalin
