# Supplementary material for: Urinary Neutrophil Gelatinase-Associated Lipocalin (NGAL) and proteinuria predict severity of acute kidney injury in Puumala virus infection
Source: BMC Infect Dis. 2015 Oct 27;15:464. doi: 10.1186/s12879-015-1180-9 (PMC4621931; doi:10.1186/s12879-015-1180-9)
Supplement: Additional file 1: — NE-NGAL BMC supp. data rev 2.0.pptx (PPTX 161 kb) [file 12879_2015_1180_MOESM1_ESM.pptx]

## Slide 1
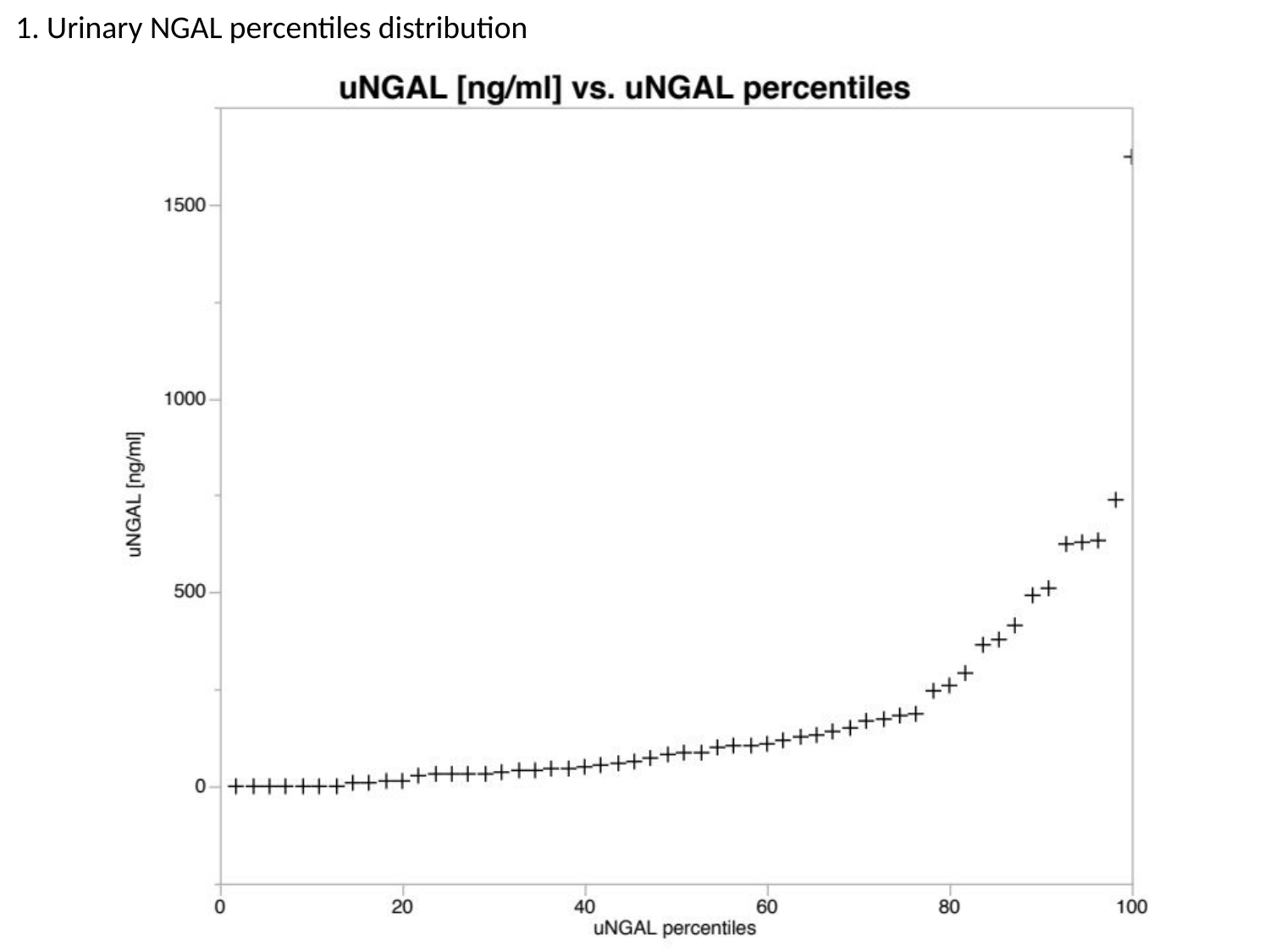

1. Urinary NGAL percentiles distribution

## Slide 2
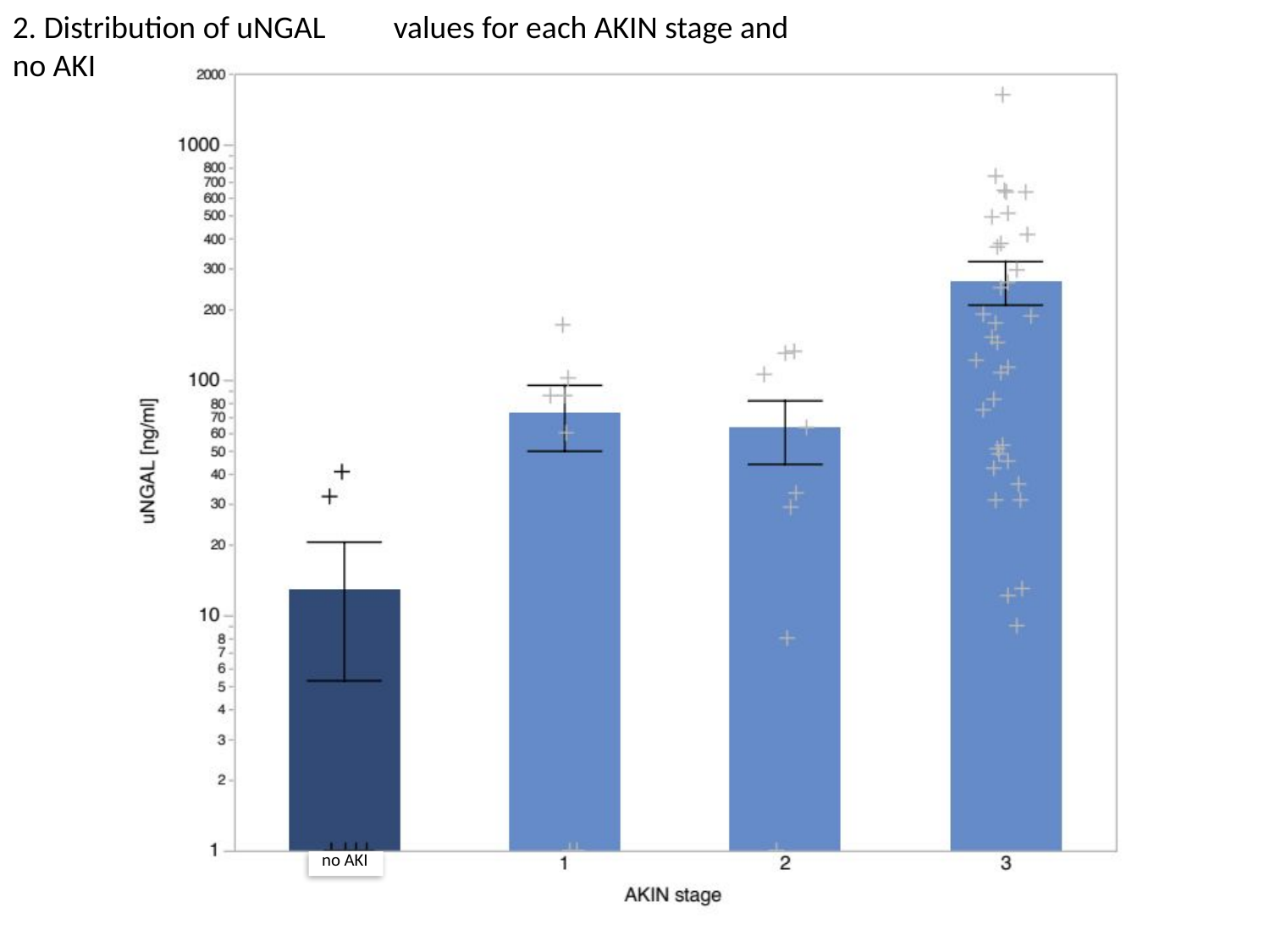

2. Distribution of uNGAL	values for each AKIN stage and no AKI
no AKI

## Slide 3
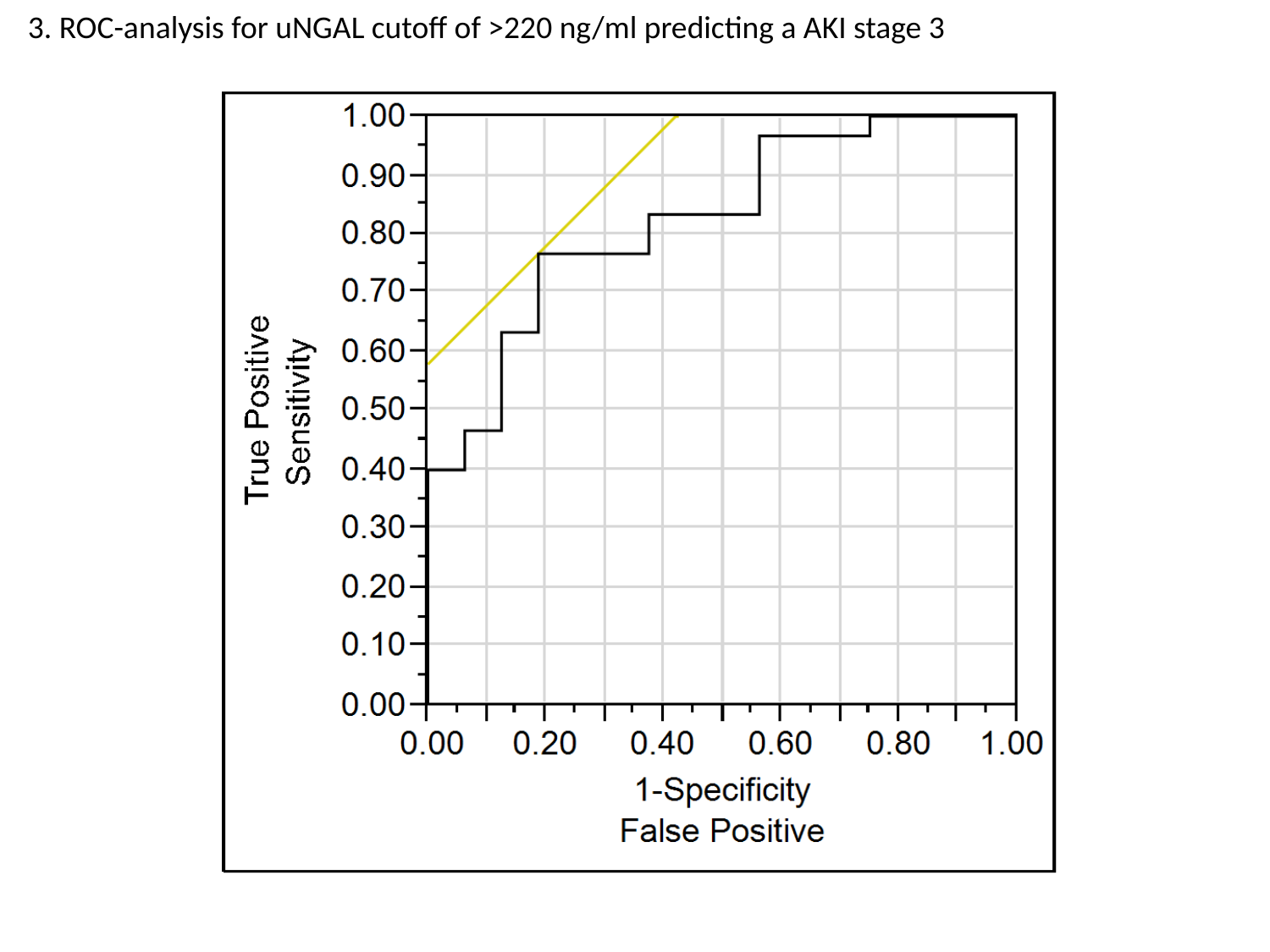

3. ROC-analysis for uNGAL cutoff of >220 ng/ml predicting a AKI stage 3
